# Efficacy and safety of anti-vascular endothelial growth factor agents in the treatment of primary pterygium

**DOI:** 10.3389/fmed.2023.1166957

**Published:** 2023-05-23

**Authors:** Bowen Zhang, Xingmei Dong, Yi Sun

**Affiliations:** ^1^Department of Operating Room, The First Affiliated Hospital of Guangzhou Medical University, Guangzhou, China; ^2^Department of Pathology, The Second Affiliated Hospital of Guangzhou University of Chinese Medicine (Guangdong Provincial Hospital of Chinese Medicine), Guangzhou, China; ^3^Department of Ophthalmology, Third Affiliated Hospital of Sun Yat-sen University, Guangzhou, China

**Keywords:** anti-vascular endothelial growth factor, primary pterygium, efficacy, safety, meta-analysis

## Abstract

**Purpose:**

To further evaluate the efficacy and safety of anti-vascular endothelial growth factor (VEGF) agents in management of primary pterygium.

**Methods:**

Randomized controlled trials (RCTs) in databases of PubMed, Web of Science, Embase, and the Cochrane Central Register of Controlled Trials were searched from inception to September 2022. Recurrences and complications were evaluated as the pooled risk ratio (RR) and 95% confidence interval (CI) using random-effects model.

**Results:**

In total of 1,096 eyes in 19 RCTs were included. Anti-VEGF agents statistically decreased recurrence rate of pterygium following surgery (RR 0.47, 95% CI 0.31–0.74, *P* < 0.001). Subgroup analysis showed that anti-VEGF as an adjunct to bare sclera (RR 0.34, 95% CI 0.13–0.90, *P* = 0.03) and conjunctival autograft (RR 0.50, 95% CI 0.26–0.96, *P* = 0.04) statistically reduced recurrence rate, while the effect was not favorable for conjunctivo-limbo autograft (RR 0.99, 95% CI 0.36–2.68, *P* = 0.98). Anti-VEGF agents statistically decreased recurrence in White patients (RR 0.48, 95% CI 0.28–0.83, *P* = 0.008), while didn't in Yellow patients (RR 0.43, 95% CI 0.12–1.47, *P* = 0.18). Both topical (RR 0.19, 95% CI 0.08–0.45, *P* < 0.001) and subconjunctival anti-VEGF agents (RR 0.64, 95% CI 0.45–0.91, *P* = 0.01) had a positive influence on recurrence. There was no statistically significant difference in complications between the groups (RR 0.80, 95% CI 0.52–1.22, *P* = 0.29).

**Conclusions:**

As adjuvant treatment, anti-VEGF agents statistically reduced the recurrence following pterygium surgery, especially among White patients. Anti-VEGF agents were well tolerated without increased complications.

## 1. Introduction

As a frequent ocular disease, pterygium is the growth of the fibrovascular conjunctiva tissue into the cornea (1). Surgery is often required when pterygium causes blur of vision, ocular mobility restriction, or even cosmetic dissatisfaction (2). However, the main concern about the surgery is the high level of recurrence, with about 1.9–8% in conjunctival autograft (3), 38–88% in bare sclera, (4) and 0–17% in limbal conjunctival autograft (5). Therefore, many adjuvant treatments, including 5-FU (6), mitomycin C (7), and ciclosporin A (8, 9), have been developed trying to diminish recurrence.

Among the risk factors responsible for pterygium growth, vascular endothelial growth factor (VEGF) plays an important role (10). Compared to normal conjunctiva, a higher level of VEGF was presented in pterygium (11). Consequently, several anti-VEGF agents, mainly bevacizumab, were afterward administered in treating pterygium. Dozens of randomized controlled trials (RCTs) on the safety and efficacy of bevacizumab in pterygium showed inconsistent conclusions (12–30). Although our previous meta-analysis (2) and a recently published paper by Zhang (31) revealed that bevacizumab reduced recurrence after pterygium surgery, the finding wasn't conclusively supported by researches thereafter and the data just focused on bevacizumab. Some other anti-VEGF agents, including ranibizumab (32–35), conbercept (36), and aflibercept (37) also showed different efficacy in management of pterygium. Overall, the current evidence does not convincingly support the use of anti-VEGF in pterygium surgery (10). Whether anti-VEGF drugs can reduce recurrence following pterygium surgery remains unanswered.

The current study is therefore designed to further evaluate the influence of all anti-VEGF agents on primary pterygium in terms of recurrence and complication.

## 2. Methods

### 2.1. Search strategy

The study was conducted in accordance with the preferred reporting items for systematic reviews and meta-analyses (PRISMA) guidelines. Databases of PubMed, Web of Science, Embase, and the Cochrane Central Register of Controlled Trials (CENTRAL) were searched from inception to September 2022. Relevant keywords and medical subject heading (MeSH) terms were used, which included: (1) “anti-vascular endothelial growth factor” OR “anti-VEGF” OR “Bevacizumab” OR “Ranibizumab” OR “Conbercept” OR “aflibercept”; (2) “pterygium” OR “pterygia”. Details of the literature searching were demonstrated in the supplemental Search Strategy file. Endnote software was used. Titles and/or abstracts were screened to subtract evidently irrelevant literatures. Full texts were estimated for qualification. To discern studies not found by the electronic searches, we performed a manual search by checking the reference lists of all preliminarily enrolled studies. Language limitation was not utilized.

### 2.2. Inclusion and exclusion criteria

The qualified articles should fulfill the inclusion criteria: (1) Participants: patients with primary pterygium; (2) Intervention: topical or subconjunctival anti-VEGF agents, despite operation or not. The dose of anti-VEGF agents or follow-up duration were not restricted; (3) Comparison: anti-VEGF agents and control; (4) Outcomes: recurrence rate and/or complication; (5) Study type: RCT. RCTs were excluded if the raw data was unavailable for extraction.

### 2.3. Outcome measurements

The recurrence rates and complications were the primary outcome measurements. Fibrovascular growth developing cross the cornea was diagnosed as recurrence. The number of recurrences was calculated at the endpoint of the follow-up. The number of complications such as subconjunctival hemorrhage, corneal dellen, and systemic complications during the follow-up in each study was counted.

### 2.4. Data extraction

Two authors (BWZ and XMD) independently performed the data extraction. The information collected from each study included the first author's last name, year of publication, location, sample size, type of anti-VEGF, route of administration, age, follow-up duration, and treatment method. Discrepancies between the authors were resolved by discussion to obtain a consensus.

### 2.5. Risk of bias assessment

According to the methods represented in the Cochrane Handbook for Systematic Reviews of Interventions 5.3, two authors (BWZ and XMD) independently assessed the risk of bias in each study. The authors reviewed each study and rated “low”, “high”, or “unclear” to the following items: (1) selection bias (Was there sufficient generation of the allocation concealment and randomization sequence?); (2) performance and detection bias (Was there blinding of personnel, participants, and outcome assessors?); (3) attrition bias (Were there incomplete outcome data and how to deal with this?); (4) reporting bias (Was there evidence of reporting outcome selectively?); and (5) other sources of bias (Were there any other potential threats to validity?). Any conflict was discussed until agreement was reached.

### 2.6. Statistical analysis

Statistical analyses were administered using RevMan 5.3 (The Cochrane Collaboration, Copenhagen, Denmark). The recurrence rates and complications were considered as dichotomous variables, which were measured as risk ratio (*RR*) with a 95% confidence interval (CI). It was assumed that heterogeneity still existed because of the diversity in clinical characteristics and differences in sample size among the studies, even when no statistical significance was observed. Thus, random-effects model was used to pool the data. Statistical heterogeneity was evaluated by calculating an *I*^2^ statistic and a Cochran Q statistic. Subgroup analysis were conducted to further assess the influence of the following factors on the recurrence: (a) topical or subconjunctival injection of anti-VEGF agents; (b) types of surgery; (c) races of patients. Sensitivity analysis was performed by leaving studies one-by-one to evaluate the stability of the results. Publication biases were detected according to symmetry in funnel plots. A *P* value <0.05 was considered statistically significant.

## 3. Results

### 3.1. Literature search

**Figure 1:**
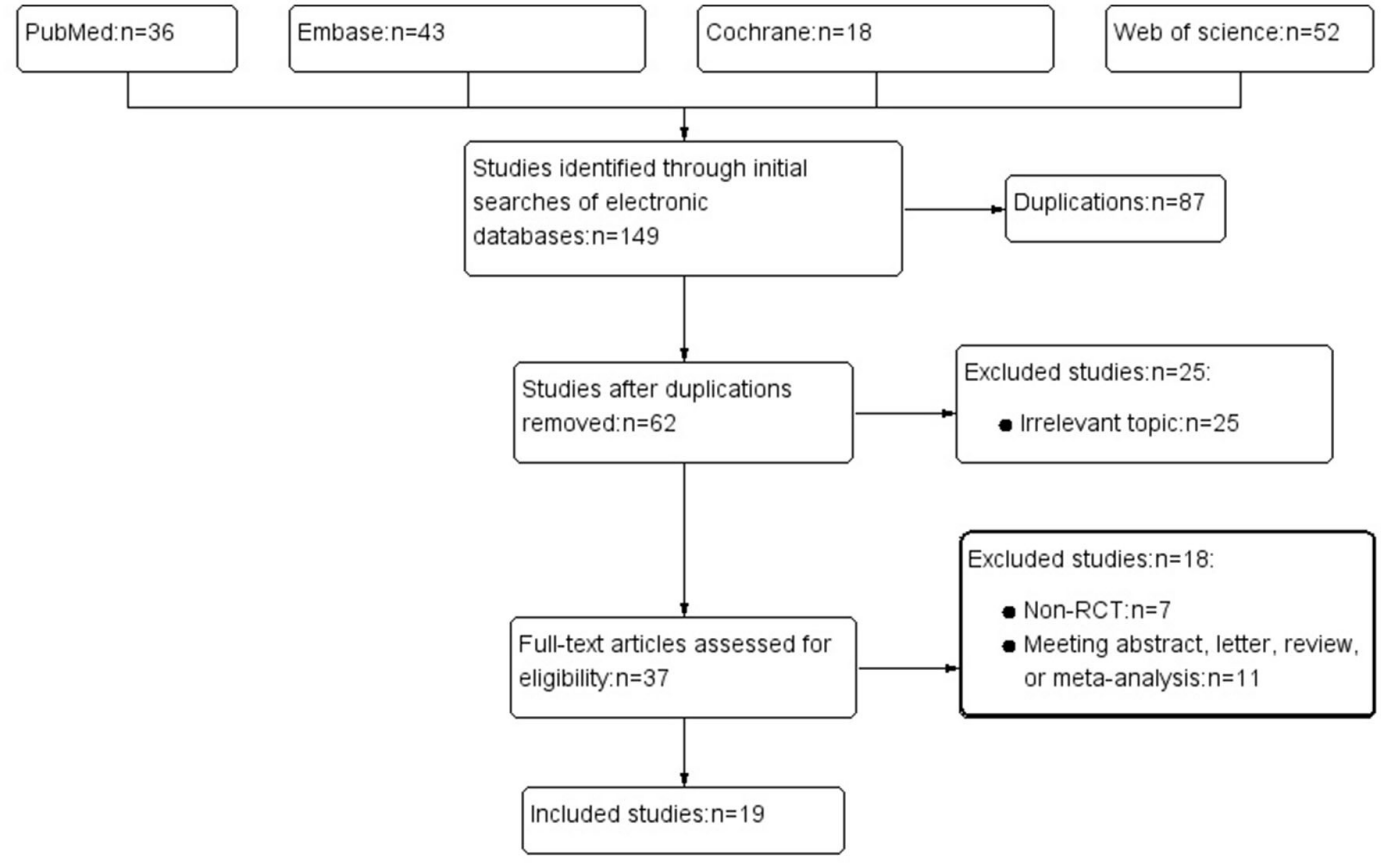
Flow diagram for the literature search and selection process.

Process of literature search was summarized in Figure 1. Initially, 149 articles were enrolled. The abstracts of the left studies were screened following the removed duplications. A 37 articles with probably related topic were further reviewed in full texts. In total of 19 RCTs were finally included in the study.

### 3.2. Characteristics and quality assessment of the eligible studies

Characteristics of the enrolled studies were shown in Table 1. A total of 19 RCTs were included (12–26, 30, 32, 36, 38). There were 18 English articles and 1 Chinese articles. In total of 1,096 eyes were included: 570 in the anti-VEGF group whereas 526 in the control group. Based on Cochrane Handbook for Systematic Reviews of Interventions 5.3, quality assessment was performed. The risks of bias for the studies were listed in Supplementary Figures S1A, B.

**Table 1:** Characteristics of the enrolled randomized clinical trials.

**References**	**Location**	**No. of eyes (Anti-VEGF/Control)**	**Type of anti-VEGF**	**Route of administration**	**Mean age (Anti-VEGF/Control, years)**	**Follow-up (months)**	**Treatment method**
Razeghinejad et al. (21)	Iran	15/15	Bevacizumab	Subconjunctival	45.8/41.6	8 vs. 7.4	Conjunctival autograft
Banifatemi et al. (16)	Iran	22/22	Bevacizumab	Subconjunctival	41.95/44.13	1	Conjunctival autograft
Enkvetchakul et al. (15)	Thailand	34/40	Bevacizumab	Subconjunctival	51.5/49	6	Non-surgery
Shenasi et al. (26)	Iran	33/33	Bevacizumab	Subconjunctival	58.67/55.94	9	Bare sclera
Shahin et al. (20)	Egypt	20/21	Bevacizumab	Subconjunctival	58.40/57.58	8	Conjunctivo-limbal autograft
Xu et al. (38)	China	40/40	Bevacizumab	Subconjunctival	44/41	12	Conjunctivo-limbal autograft
Nava-Castaneda et al. (23)	Mexico	33/16	Bevacizumab	Subconjunctival	48.75/47.8	12	Conjunctival autograft
Karalezli et al. (17)	Turkey	42/46	Bevacizumab	Topical	58.82/53.04	29.3 vs. 28.5	Conjunctival autograft
Razeghinejad et al. (25)	Iran	20/21	Bevacizumab	Subconjunctival	41.95/44.13	6	Conjunctival autograft
Ozsutcu et al. (24)	Turkey	30/30	Bevacizumab	Subconjunctival	43.25/41.68	9	Conjunctival autograft
Kasetsuwan et al. (22)	Thailand	12/10	Bevacizumab	Topical	50.7/59.3	3	Bare sclera
Hwang et al. (30)	Korea	36/33	Bevacizumab	Topical	71.3/73.4	6	Bare sclera
Singh et al. (14)	India	30/30	Bevacizumab	Subconjunctival	37.33	3	Conjunctival autograft
Bekibele et al. (12)	Nigeria	26/27	Bevacizumab	Subconjunctival	49.2/52.0	18.35	Conjunctival autograft
Motarjemizadeh et al. (13)	Iran	60/30	Bevacizumab	Topical	39.47/40.97	12	Bare sclera
Nuzzi et al. (19)	Italy	42/41	Bevacizumab	Subconjunctival	52.39/54.02	6	Bare sclera
Mohamed et al. (18)	Egypt	22/18	Bevacizumab	Subconjunctival	31–58	1	Conjunctival autograft
Zhang et al. (36)	China	48/48	Conbercept	Subconjunctival	60.13/61.02	6	Bare sclera/Conjunctival autograft
Mandalos et al. (32)	Greece	5/5	Ranibizumab	Subconjunctival	66.6	/	Bare sclera

VEGF, vascular endothelial growth factor.

### 3.3. Meta-analysis

**Figure 2:**
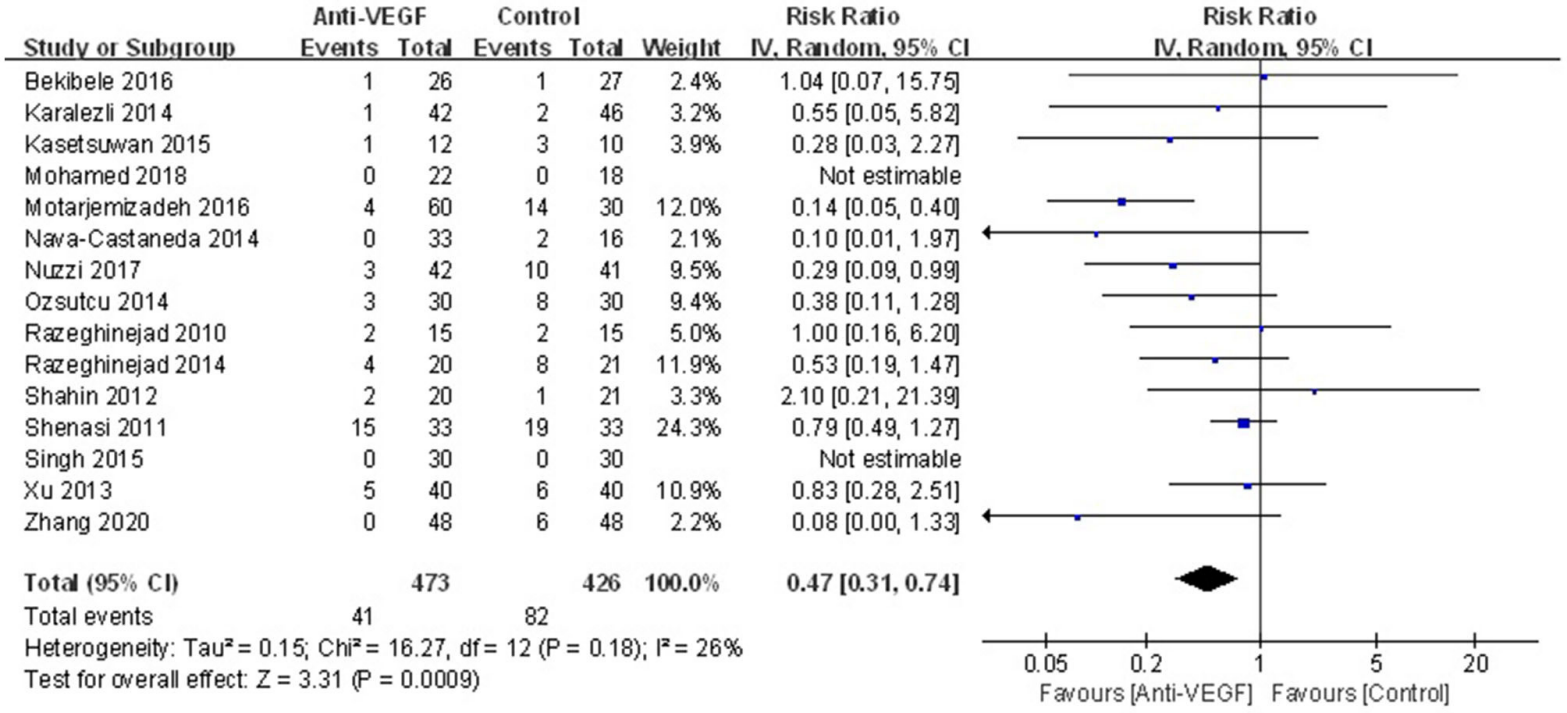
Forest plot for the overall recurrence of anti-VEGF drugs for primary pterygium.

**Figure 3:**
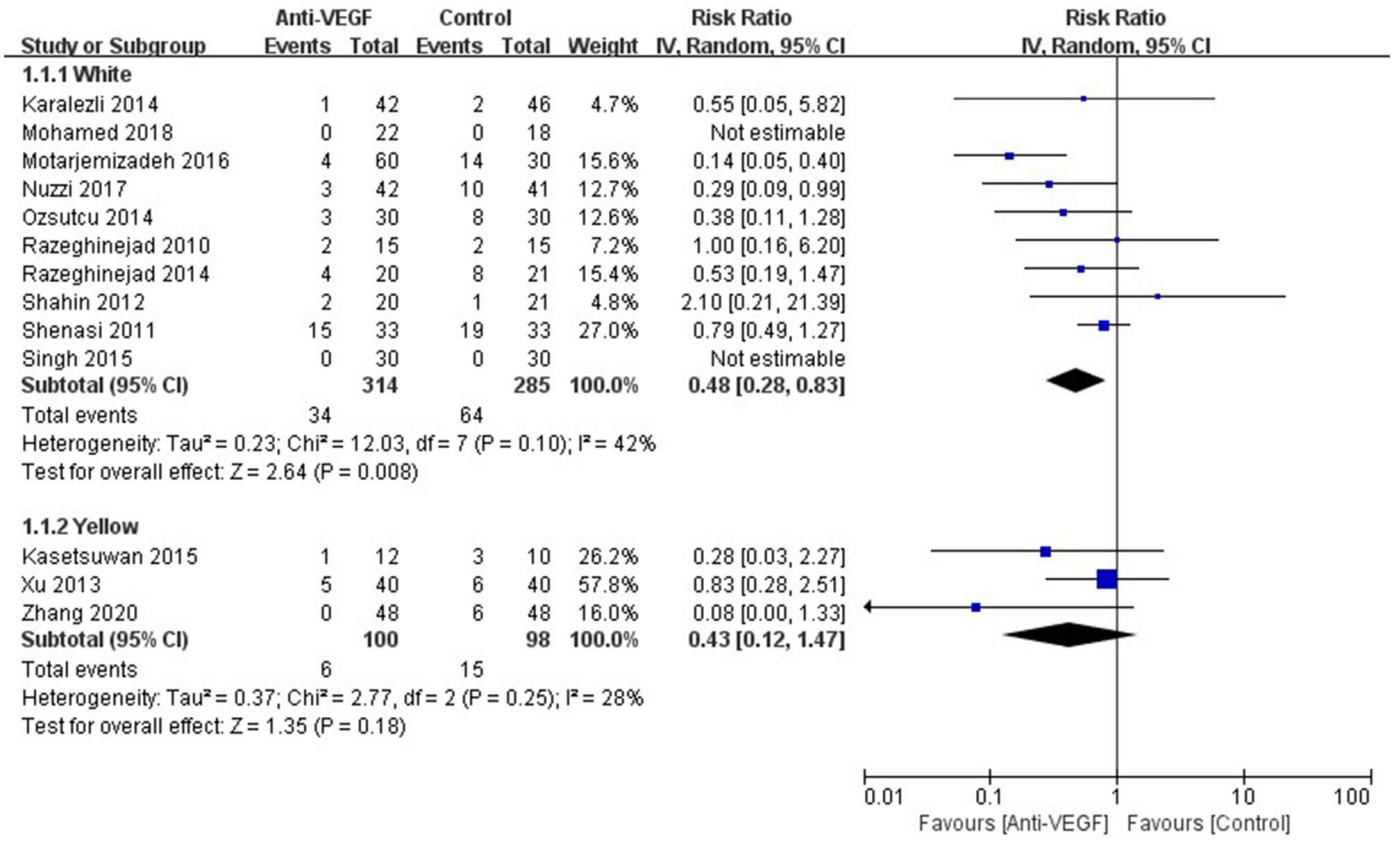
Forest plot for the recurrence of anti-VEGF drugs for primary pterygium which was stratified by races.

Recurrence was reported in 15 studies. The definitions of recurrence in the included RCTs were summarized in Table 2. Overall recurrence in the current study was presented in Figure 2. The results showed that anti-VEGF agents significantly decreased recurrence (RR 0.47, 95% CI 0.31–0.74, *P* < 0.001; *P*_*heterogeneity*_ = 0.18, *I*^2^ = 26%). The sensitivity analysis for the recurrence was stable. Subgroup analysis stratified by races indicated that anti-VEGF agents statistically decreased recurrence in White patients (RR 0.48, 95% CI 0.28–0.83, *P* = 0.008; *P*_*heterogeneity*_ = 0.10, *I*^2^ = 42%), while didn't in Yellow patients (RR 0.43, 95% CI 0.12–1.47, *P* = 0.18; *P*_*heterogeneity*_ = 0.25, *I*^2^ = 28%) (Figure 3). Subgroup analysis in terms of the surgery options showed that bare sclera (RR 0.34, 95% CI 0.13–0.90, *P* = 0.03; *P*_*heterogeneity*_ = 0.02, *I*^2^ = 71%) and conjunctival autograft (RR 0.50, 95% CI 0.26–0.96, *P* = 0.04; *P*_*heterogeneity*_ = 0.82, *I*^2^ = 0) statistically reduced recurrence, while conjunctivo-limbo autograft did not (RR 0.99, 95% CI 0.36–2.68, *P* = 0.98; *P*_*heterogeneity*_ = 0.48, *I*^2^ = 0) (Supplementary Figure S2A). Subgroup analysis based on the administration of anti-VEGF agents demonstrated that both topical (RR 0.19, 95% CI 0.08–0.45, *P* < 0.001; *P*_*heterogeneity*_ = 0.55, *I*^2^ = 0) and subconjunctival application (RR 0.64, 95% CI 0.45–0.91, *P* = 0.01; *P*_*heterogeneity*_ = 0.49, *I*^2^ = 0) could significantly reduce recurrence (Supplementary Figure S2B).

**Table 2:** Definition of recurrence of pterygium in the enrolled studies.

**References**	**Definition of recurrence**
Razeghinejad et al. (21)	Fibrovascular tissue extending more than 1.5 mm across limbus
Shenasi et al. (26)	Fibrovascular growth crossing limbus and extending over the cornea to any distance
Shahin et al. (20)	4 grades classified
Xu et al. (38)	Fibrovascular tissue invading cornea
Nava-Castaneda, A et al. (23)	4 grades classified
Karalezli et al. (17)	Fibrovascular growth passing the corneal limbus by more than 1 mm
Razeghinejad et al. (25)	More than 1.5 mm of fibrovascular tissue overgrowth on cornea and any fibrovascular tissue crossing limbus
Ozsutcu et al. (24)	Any fibrovascular growth of conjunctival tissue extending more than 1.5 mm across limbus
Kasetsuwan et al. (22)	4 grades classified
Singh et al. (14)	4 grades classified
Bekibele et al. (12)	Growth of fibrovascular tissue 1 mm or more into cornea
Motarjemizadeh et al. (13)	New vessels or fibrovascular connective tissues crossing corneal limbus
Nuzzi et al. (19)	Growth of fibrovascular tissue extending more than 1 mm across the limbus
Mohamed et al. (18)	No definition
Zhang et al. (36)	4 grades classified

**Figure 4:**
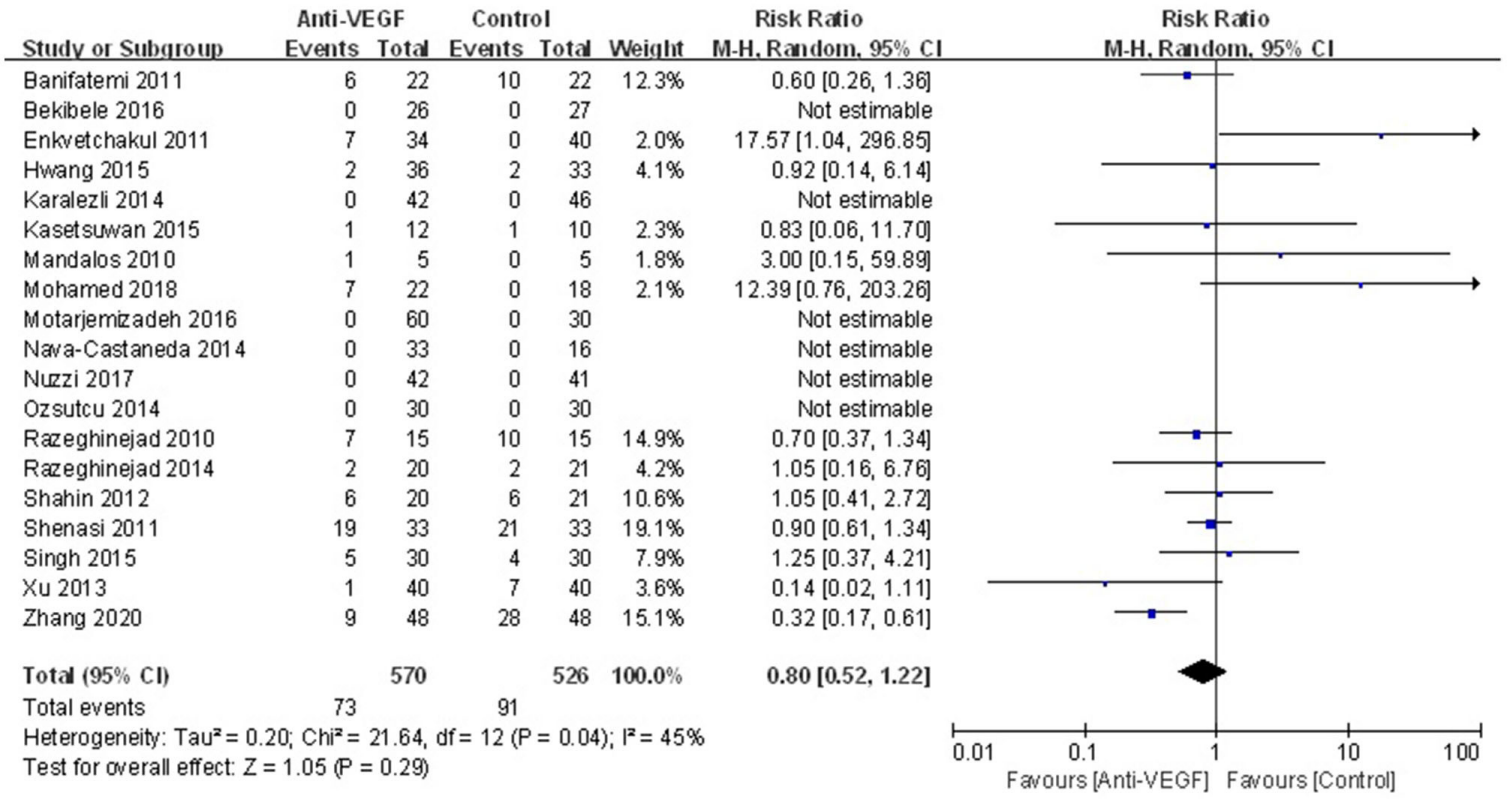
Forest plot for the overall complications associated with anti-VEGF drugs for primary pterygium.

Complications were reported in 19 RCTs. There was no statistically significant difference in complications between anti-VEGF group and control group (RR 0.80, 95% CI 0.52–1.22, *P* = 0.29; *P*_*heterogeneity*_ = 0.04, *I*^2^ = 45%) (Figure 4). Especially, rate of subconjunctival hemorrhage between the both groups was not statistically different (RR 1.44, 95% CI 0.76–2.71, *P* = 0.27; *P*_*heterogeneity*_ = 0.41, *I*^2^ = 3%) (Supplementary Figure S3). The sensitivity analysis for the complications was stable.

Funnel plots displayed insignificant publication biases for recurrence and complication (Supplementary Figures S4A, B).

## 4. Discussion

The present study is a comprehensive analysis on the efficacy and safety of anti-VEGF adjuvant treatments for primary pterygium, which includes not only the commonly used bevacizumab, but also ranibizumab and conbercept. Results from the study found out that, anti-VEGF agents, regardless of topical or subconjunctival administration, were statistically effective for reducing recurrence following pterygium excision by bare sclera or conjunctival autograft, while the complications were not increased.

Although there are several meta-analyses about the effect of anti-VEGF drugs on pterygium, all of them focused on bevacizumab (2, 3, 31, 39–42), probably due to its lower cost. The efficacy of other newer anti-VEGF adjuncts, for instance, conbercept and ranibizumab, was not involved in any meta-analysis. This is an important reason why the current study was carried out. The most recent meta-analysis regarding the efficacy of bevacizumab on pterygium was conducted by Zhang et al. (31), the study type of which was RCTs. But among the included studies, two are retrospective analysis instead of RCTs. Thus, its conclusion is in question. Compared to our previous meta-analysis on the relevant topic in 2018 (2), 4 RCTs are added thereafter to the current study. So, it is more likely for us to supply newer and definite evidence for the unresolved issue.

Subgroup analysis showed that compared to control, anti-VEGF in combination with conjunctivo-limbal autograft didn't statistically reduce recurrence rate. The reason probably lies in the limited power due to only 2 involved studies and the trivial efficacy of anti-VEGF agents compared to corneal limbus stem cells. Only 1 RCT studied the efficacy of anti-VEGF agent on recurrent pterygia, making it difficult to pool the data. Therefore, we didn't reanalyze the effect of anti-VEGF agents in recurrent pterygia. It is believed that anti-VEGF agents were not as much effective as in primary pteryium than in recurrent pteriugium because these drugs affect neovascularization rather than old and organized vessels (43).

An interesting finding is that anti-VEGF agents were effective in reducing recurrence among White patients. The reasons for that remain unknown. Future researches focusing on the variation of VEGF among pterygium patients with different races may partly reveal the underlying cause.

Except bevacizumab, there were few studies on the effect of other anti-VEGF agents in pterygium, probably due to the higher costs. Therefore, the sample size was also usually small, which might draw inconclusive results. For example, regarding ranibizumab, subconjunctival ranibizumab had no effect on the extent of vascularization of primary pterygium (32), but it appeared to arrest growth in early recurrent pterygium (33). In another study (34), the recurrence was 3/10 among primary pterygium patients underwent surgery with subconjunctival ranibizumab. Regarding conbercept, there was only one study showing that multiple subconjunctival conbercept injections were effective and safe (36). As for aflibercept, several non-RCT studies indicated that aflibercept was a safe method of reducing inflammation, fibrovascular proliferation, and recurrence (37, 44). Therefore, more well-designed RCTs with large sample size are needed to further determine whether ranibizumab, conbercept, and aflibercept actually decrease pterygium recurrence, and which one is superior.

Ocular complications including subconjunctival hemorrhage, conjunctival cyst, graft edema, corneal epithelial defect, aseptic scleritis and infections, as well as the systematic complications were mainly evaluated. The pooled results showed that anti-VEGF drugs were not associated with more complications compared to controls, indicating their safety. It is consistent with many studies on bevacizumab (2, 31, 42). Subconjunctival administration especially requiring more frequent injection probably cause more side effects than topical administration. Subconjunctival hemorrhage was particularly concerned. We believe it is not appropriate to consider subconjunctival hemorrhage as a complication in the study by Hwang et al. (30) because bevacizumab was only used topically.

Several limitations must be mentioned in the study. First, the definition of recurrence varied among studies. According to Tseng's criteria (45), Grade IV, which is defined as fibrovascular tissue extending past the limbus, is the true recurrence. But different criteria were used in the included primary studies. Second, studies showed that half of recurrences probably occur within 4 months, and 97% probably occur within 12 months (46). Therefore, follow-up of 1 year or over is necessary. Most of the included studies reported recurrence with follow-up of <1 year, which might underestimate the true recurrence. Third, the optimal route and dosage of anti-VEGF drugs, remains unanswered. Therefore, caution is required in the interpretation and further well-designed studies are still needed.

In conclusion, the study showed that topical or subconjunctival anti-VEGF agents could significantly decrease recurrence following pterygium excision by bare sclera or conjunctival autograft, while the complications were not increased. Anti-VEGF agents were especially effective in reducing recurrence among White patients. Anti-VEGF is an effective and safe method in the management of primary pterygium.

## Data availability statement

The original contributions presented in the study are included in the article/Supplementary material, further inquiries can be directed to the corresponding author.

## Author contributions

YS: conception, design, and data interpretation. BZ and XD: collection and assembly of data and data analysis. All authors: manuscript writing and final approval of manuscript.
