# Supplementary figures and images for: Efficacy and safety of anti-vascular endothelial growth factor agents in the treatment of primary pterygium
Source: Front Med (Lausanne). 2023 May 23;10:1166957. doi: 10.3389/fmed.2023.1166957 (PMC10242018; doi:10.3389/fmed.2023.1166957)

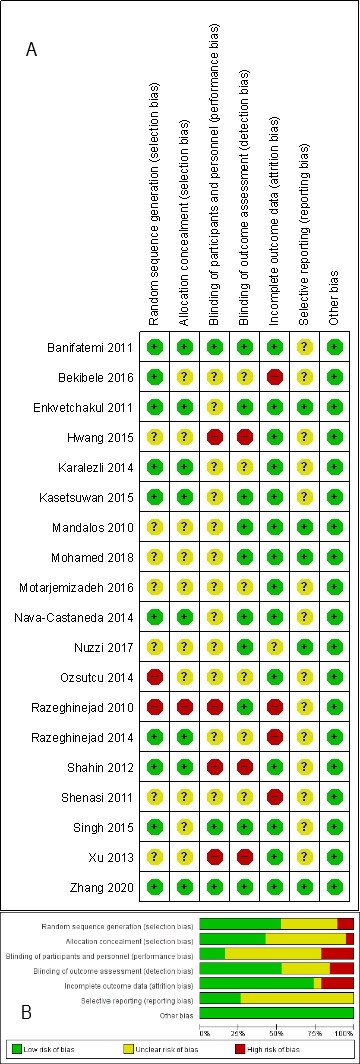

Supplement: Supplementary file 1 [file Image_1.JPEG]

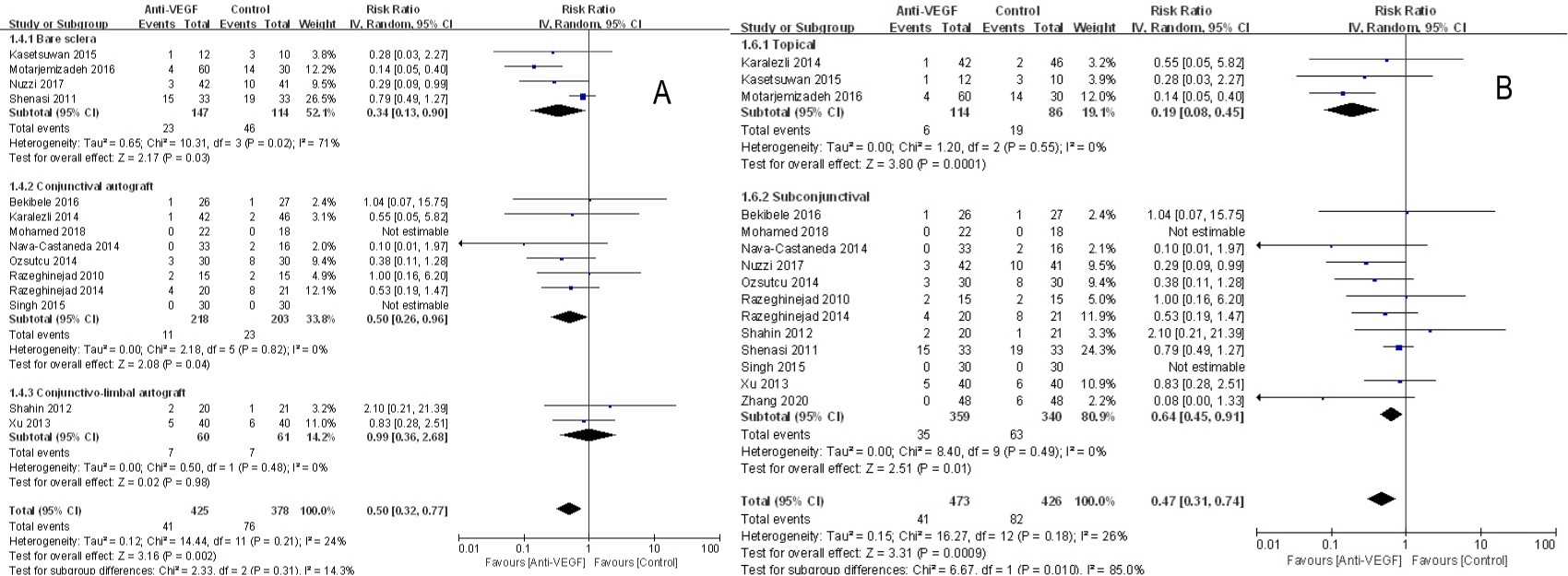

Supplement: Supplementary file 2 [file Image_2.JPEG]

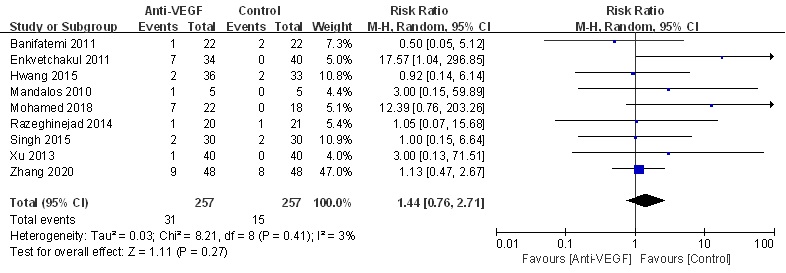

Supplement: Supplementary file 3 [file Image_3.JPEG]

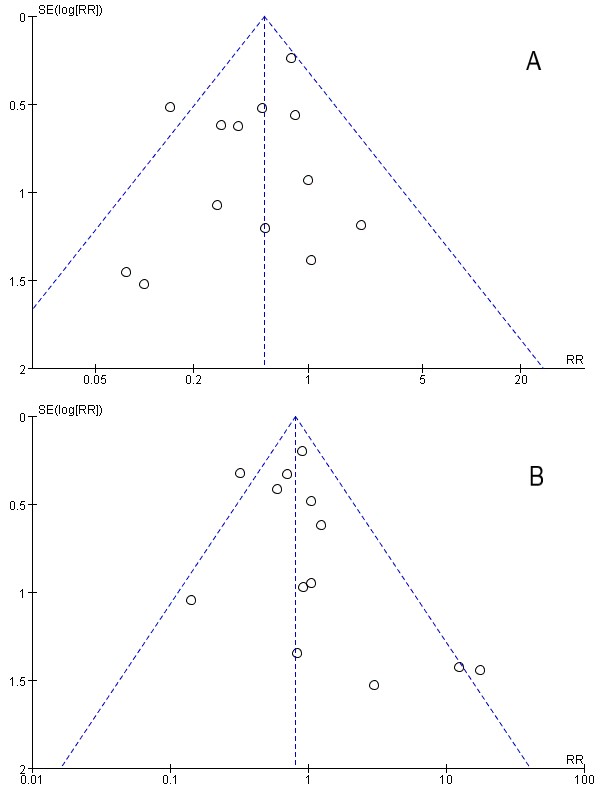

Supplement: Supplementary file 4 [file Image_4.JPEG]
