# Supplementary material for: Efficacy and safety of anti-vascular endothelial growth factor agents in the treatment of primary pterygium
Source: Front Med (Lausanne). 2023 May 23;10:1166957. doi: 10.3389/fmed.2023.1166957 (PMC10242018; doi:10.3389/fmed.2023.1166957)
Supplement: Supplementary file 5 [file Data_Sheet_1.PDF]

Search strategy for Pubmed:

#1 "Single-Blind Method"[Mesh] OR "Double-Blind Method"[Mesh] OR "Randomized Controlled Trials as Topic"[Mesh] OR "Randomized Controlled Trial" [Publication Type] OR "Intention to Treat Analysis"[Mesh] OR "Controlled Clinical Trials as Topic"[Mesh] OR "Clinical Trials as Topic"[Mesh] OR "Clinical Trial" [Publication Type] OR randomized controlled trial[Publication Type]

#2 "random*"[Text Word] OR allocation[Text Word] OR "random allocation"[Text Word] OR placebo[Text Word] OR single blind[Text Word] OR double blind[Text Word] OR "randomized controlled trial*"[Text Word] OR RCT[Text Word]

#3 #1 OR #2

#4 bevacizumab OR Avastin OR conbercept OR ranibizumab OR Lucentis OR aflibercept OR Eylea OR (anti-vascular Endothelial Growth Factor)

#5 pteryg* OR primary pteryg*

#6 #3 and #4 and #5

Search strategy for web of science:

#1 TS=("random*" OR allocation OR "random allocation" OR placebo OR single blind OR single blind method OR double blind OR double blind method OR "randomized controlled trial*" OR "randomised controlled trial*" OR "RCT" OR "clinical trial*")

#2 TS= (bevacizumab OR Avastin OR conbercept OR ranibizumab OR Lucentis OR aflibercept OR Eylea OR (anti-vascular Endothelial Growth Factor))

#3 TS= (pteryg* OR primary pteryg*)

#4 #1 AND #2 AND #3

Search strategy for EMBASE:

#1 'randomization'/exp OR 'placebo'/exp OR 'placebo effect'/exp OR 'single blind procedure'/exp OR 'double blind procedure'/exp OR 'randomized controlled trial'/exp OR 'randomized controlled trial (topic)'/exp OR 'controlled clinical trial'/exp OR 'controlled clinical trial (topic)'/exp OR 'clinical trial'/exp OR 'clinical trial (topic)'/exp

#2 random*:ab,ti OR allocation:ab,ti OR "random allocation":ab,ti OR placebo:ab,ti OR single blind:ab,ti OR double blind:ab,ti OR randomised controlled trial*:ab,ti OR randomized controlled trial*:ab,ti OR RCT:ab,ti OR clinical trial*:ab,ti

#3 #1 OR #2

#4 bevacizumab:ab,ti OR Avastin:ab,ti OR conbercept:ab,ti OR ranibizumab:ab,ti OR Lucentis:ab,ti OR aflibercept:ab,ti OR Eylea:ab,ti OR (anti-vascular Endothelial Growth Factor):ab,ti

#5 pteryg*:ab,ti OR primary pteryg*:ab,ti

#6 #3 AND #4 AND #5

Search strategy for Cochrane Library:

#1 "random*" or allocation or "random allocation" or placebo or single blind or double blind or "randomized controlled trial*" or RCT or "clinical trial*"

#2 randomized controlled trial:pt or clinical trial:pt

#3 bevacizumab or Avastin OR conbercept OR ranibizumab OR Lucentis OR aflibercept OR Eylea OR (anti-vascular Endothelial Growth Factor)

#4 pteryg* or primary pteryg*

#5 #1 and #2 and #3 and #4
